# Active and resting motor threshold are efficiently obtained with adaptive threshold hunting

**DOI:** 10.1371/journal.pone.0186007

**Published:** 2017-10-05

**Authors:** Christelle B. Ah Sen, Hunter J. Fassett, Jenin El-Sayes, Claudia V. Turco, Mahdiya M. Hameer, Aimee J. Nelson

**Affiliations:** Department of Kinesiology, McMaster University, Hamilton, Ontario, Canada; University of Ottawa, CANADA

## Abstract

Transcranial magnetic studies typically rely on measures of active and resting motor threshold (i.e. AMT, RMT). Previous work has demonstrated that adaptive threshold hunting approaches are efficient for estimating RMT. To date, no study has compared motor threshold estimation approaches for measures of AMT, yet this measure is fundamental in transcranial magnetic stimulation (TMS) studies that probe intracortical circuits. The present study compared two methods for acquiring AMT and RMT: the Rossini-Rothwell (R-R) relative-frequency estimation method and an adaptive threshold-hunting method based on maximum-likelihood parameter estimation by sequential testing (ML-PEST). AMT and RMT were quantified via the R-R and ML-PEST methods in 15 healthy right-handed participants in an experimenter-blinded within-subject study design. AMT and RMT estimations obtained with both the R-R and ML-PEST approaches were not different, with strong intraclass correlation and good limits of agreement. However, ML-PEST required 17 and 15 fewer stimuli than the R-R method for the AMT and RMT estimation, respectively. ML-PEST is effective in reducing the number of TMS pulses required to estimate AMT and RMT without compromising the accuracy of these estimates. Using ML-PEST to estimate AMT and RMT increases the efficiency of the TMS experiment as it reduces the number of pulses to acquire these measures without compromising accuracy. The benefits of using the ML-PEST approach are amplified when multiple target muscles are tested within a session.

## Introduction

Transcranial magnetic stimulation (TMS) is commonly used to non-invasively study the human motor system. One of the most fundamental measures in TMS studies is the motor threshold (MT) for a given targeted muscle. MT is considered an indicator of cortical excitability and is a critical determinant used to define TMS intensity parameters for assessing other cortical circuitry [1]. In addition, the efficient determination of MT is important with respect to safety implications, such as reducing the number of TMS pulses delivered to individuals [2]. However, it is equally important to derive AMT accurately. Therefore, achieving such accuracy is of primary importance while still minimizing the number of pulses delivered. MT estimation may be obtained when the target muscle is at rest (RMT) and during active contraction of the target muscle (AMT) [3].

The comparison of different MT assessment methods has been understudied. A recent report of the International Federation of Clinical Neurophysiology (IFCN) provided an updated review on the practical uses of TMS in clinical applications and research [3]. The guidelines outline a range of MT estimation methods, such as relative-frequency estimation (R-R) [4] and the adaptive threshold-hunting methods based on maximum likelihood parameter estimation by sequential testing (ML-PEST) [5, 6], and recommended using the adaptive threshold-tracking algorithm over other methods as it provides an accurate estimation of MT with fewer number of pulses [3]. However, the standard method used in TMS research is the R-R relative frequency estimation method [3]. No studies have examined differences in the R-R versus adaptive threshold hunting for measures of AMT, as indicated by Silbert et al. [7]. Measures of AMT may yield different results than those of RMT, since AMT corresponds to the threshold for inducing descending volleys in the fast-conducting neurons of the corticospinal tract [8]. AMT is an important consideration for research delivering repetitive TMS neuroplasticity protocols and for assessment of short-interval intracortical inhibition (SICI) and facilitation (ICF) [8, 9].

The R-R method, proposed by Rossini and colleagues in 1994, defines RMT as the lowest stimulus intensity required to elicit a MEP of ~100μV in 50% of 10–20 consecutive trials in the resting muscle [4] and was subsequently refined to ≥ 50μV in 5 out of 10 consecutive trials [10]. Similarly, AMT is defined as the lowest stimulus intensity to elicit a MEP ≥ 200μV in 5 out of 10 consecutive trials during an isometric contraction of ~10–20% MVC in the target muscle [4]. Many studies continue to use the R-R method as it was the first method of assessing MT described by the IFCN in 1994 [3, 4]. However, the R-R approach may not provide an objective mathematical computation to estimate MT [3] and may be subject to experimenter bias and variations in MT estimates [6, 11]. Additionally, the R-R method is considered time consuming due to a relatively high number of stimuli required to achieve MT estimates [3].

An alternative approach for obtaining MT involves adaptive threshold-hunting methods that are based on maximum likelihood parameter estimation by sequential testing (ML-PEST) and use a probabilistic method of estimating MT. Specifically, ML-PEST uses an S-shaped metric function to model the probabilistic nature of MT and the probability of evoking an MEP at a given stimulus intensity [6]. Using an adaptive stair-case procedure, this approach predicts a TMS intensity that yields a 50% probability of evoking an MEP, where the given predicted stimulus intensity is then selected as the intensity for the next TMS pulse [6]. ML-PEST has both a standardized TMS starting intensity (37% of the maximum stimulator output (MSO)) and employs a standardized number of pulses to achieve threshold (20 pulses in all individuals) [6]. Thus, the use of adaptive threshold-tracking algorithms may improve MT estimation over other methods since fewer pulses are required [3].

To date, few reports have compared ML-PEST and R-R methods. PEST algorithms has been shown to correctly identify RMT with fewer pulses than the R-R approach as determined through simulations [12] and in human participants [7]. The present study is the first to compare ML-PEST and R-R method for estimation of AMT. Further, similar to previous study, we compare these methods for estimation of RMT [7].

## Methods

### Participants

Fifteen healthy, right-handed individuals (21.8 ± 2.18 years, 18–27 years, 8 female) participated between February 2, 2017 and March 15, 2017. The study was advertised through posters around campus, as well as online advertising. All individuals were screened for contraindications to TMS and written consent was obtained prior to participation. The study was approved by the McMaster Research Ethics Board and conformed to the Declaration of Helsinki.

### Electromyography (EMG) recording

Electromyography (EMG) was recorded using 9mm diameter Ag-AgCl surface electrodes placed in a belly-tendon montage over the first dorsal interosseous (FDI) muscle of the right hand. A wet ground was placed around the right forearm. EMG measurements were amplified (x 1000), filtered with a band pass (20 Hz—2.5 kHz) (Intronix Technologies Corporation Model 2024F with Signal Conditioning; Intronix Technologies Corporation, Bolton, Canada), and digitized (5 kHz, Power 1401, Cambridge Electronic Design, Cambridge, UK). All EMG data was analyzed using Signal software version 6.02 (Cambridge Electronic Design, Cambridge, UK).

### Maximum Voluntary Contraction (MVC)

To determine the MVC, all participants completed three maximal isometric contractions of the right FDI against an immovable post. Each contraction persisted for 5s, followed by a 30s rest interval between trials. MVC was defined as the largest peak-to-peak amplitude of the EMG signal obtained from the three trials. Importantly, EMG was used to define MVC because it is specific to the muscle of interest (right FDI), as opposed to measures of force that may be contributed by multiple muscles. Subsequently, the voltage signal corresponding to 10% of MVC was calculated and displayed on an oscilloscope as a stationary horizontal line to act as a target during acquisition of AMT. Participants moved a second horizontal line, controlled by the FDI EMG signal, to match the position of the target line. During acquisition of AMT, all participants used their own visual feedback to maintain 10% MVC of FDI.

### Transcranial magnetic stimulation (TMS)

Single monophasic TMS pulses were delivered using a custom-built 50mm diameter figure-of-eight branding coil connected to a Magstim 200 stimulator (Magstim, Whitland, UK). The coil was positioned at 45-degree rotation in relation to the parasagittal plane to induce posterior-to-anterior current in the underlying cortex. The motor hotspot was determined with a TMS intensity ranging from 45% to 50% MSO, whereby two stimuli were systematically delivered at six varying positions across the scalp guided by Brainsight Neuronavigation using a standard anatomical MRI image. The motor hotspot was defined as the position on the scalp that yielded two consecutive MEPs with greater amplitude than the surrounding positions. The location within the left motor cortex that consistently elicited MEPs in the relaxed right FDI muscle was then defined as the motor hotspot, which was marked by digital registration using a standard MRI template via Brainsight Neuronavigation (Rogue Research, Canada). This location was used for all TMS delivery.

The R-R protocol was conducted using the Groppa modification of the “relative frequency” criterion, which states that MT is obtained at the lowest TMS intensity evoking an MEP in at least 5 out of 10 trials [3]. For the R-R protocol, the TMS stimulus intensity was set to 37% MSO as the starting intensity as performed elsewhere [7]. The 4% increment is a deviation from Groppa et al., [3]. RMT was defined as the lowest intensity required to evoke an MEP with a peak-to-peak amplitude ≥ 50μV in at least 5 out of 10 consecutive trials in the relaxed right FDI [3]. For AMT, the R-R protocol was conducted until the lowest intensity required to evoke an MEP ≥ 200μV in at least 5 out of 10 consecutive trials during isometric contraction of the FDI corresponding to 10% MVC [3]. In greater detail, for both RMT and AMT, the intensity was increased by 4% and subsequently by 1% following the trial in which six MEPs failed to reach threshold criteria, or decreased by 4% and subsequently by 1% following the trial in which five MEPs met criteria (50 μV for RMT, 200 μV for AMT). Specifically, the MSO was adjusted by 4% MSO according to the aforementioned criteria until the opposite pattern of responses was observed (i.e. decrements of 4% were performed until the first intensity that warranted an increment). Once the direction of stimulation intensity adjustment changed, 1% adjustments were made until the lowest stimulation intensity that yielded five MEPs was found. Therefore, ten trials were not necessarily delivered for each increment of MSO.

For the ML-PEST protocol, the freeware (TMS Motor Threshold Assessment Tool, MTAT 2.0) was obtained online (http://www.clinicalresearcher.org/software.html) and the option for assessment without *a priori* information was selected. The program automatically displays the subsequent TMS intensity to be delivered and begins at 37% MSO. The experimenter interacts with the program by indicating the success of a given TMS intensity: a trial is considered successful if MEP amplitude ≥ 50μV for RMT or ≥ 200μV for AMT. The software subsequently displays the next TMS intensity to be delivered. The ML-PEST algorithm was stopped after 20 stimuli which provides sufficient accuracy for the threshold estimate to comply with current safety guidelines [13].

### Experimental protocol

Both AMT and RMT were determined for the right FDI using the R-R relative frequency estimation method and the ML-PEST method. For both R-R and ML-PEST, the TMS intertrial interval was set to 6s to avoid having short intertrial intervals which may impact MEP amplitude for some subjects [14]. Using a Williams Square Design, the order of AMT and RMT acquisition with each of the two methods was randomized across participants. Three investigators participated, and only two were present at any one time. Investigator 1 carried out the R-R method to obtain AMT/RMT and was blinded to the estimates obtained using the ML-PEST method. Investigator 2 carried out the ML-PEST method and was blinded to the AMT/RMT estimates obtained using the R-R method. Investigator 3 held the coil for all TMS delivery and was not involved in determining the estimates of AMT/RMT for any method.

### Data analysis

Using IBM SPSS Statistics, outlier analysis was performed and normality was tested with the Kolmogorov-Smirnov test and all data was determined to be normally distributed. Subsequent analyses included two-tailed paired t-tests to compare the MT estimation between R-R_AMT_ and ML-PEST_AMT_, as well as R-R_RMT_ and ML-PEST_RMT_. Additionally, correlation analyses for MT estimates from the R-R and ML-PEST were performed using intraclass correlation coefficients to assess the amount of agreement between MT estimation methods, and Pearson correlations. For all analyses, the significance level was set to α ≤ 0.05. Bland-Altman plots with limits of agreement were used to understand the relation between R-R and ML-PEST methods [15].

## Results

**Fig 1:**
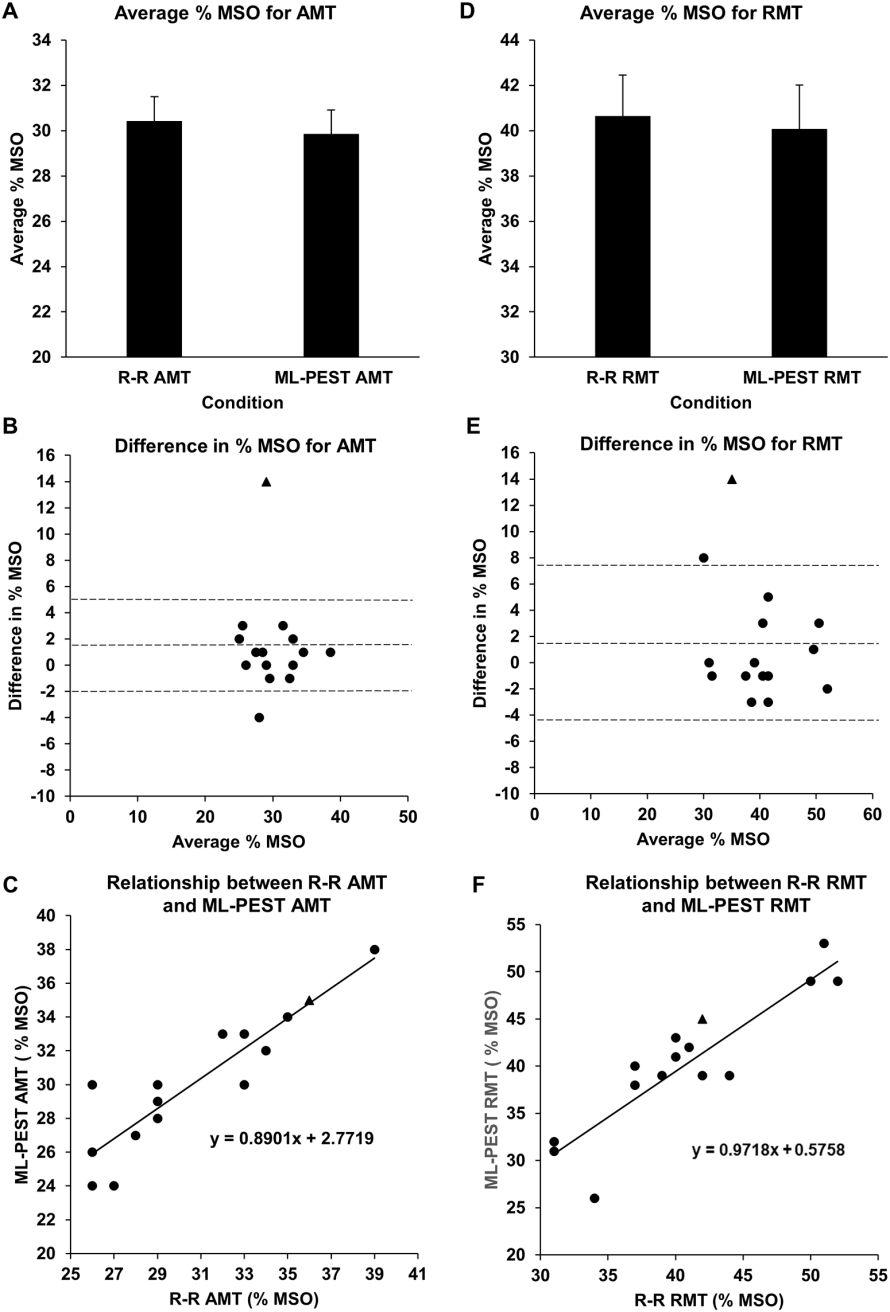
Comparison of RR and ML-PEST approach. **(**A) Comparison of the mean MSO using different testing conditions: the group-averaged MSO obtained for R-R_AMT_ and ML-PEST_AMT_ demonstrating no significant difference. (B) Limits of agreement between R-R_AMT_ and ML-PEST_AMT_ using a Bland-Altman plot demonstrating good agreement with optimal analysis for these data. (C) Scatter plot demonstrating relationship between R-R_AMT_ and ML-PEST_AMT_ also confirming similarities between estimation tools. (D) Comparison of the mean MSO using different testing conditions: the group-averaged MSO obtained for R-R_RMT_ and ML-PEST_RMT_ demonstrating no significant difference. (E) Limits of agreement between R-R_RMT_ and ML-PEST_RMT_ using a Bland-Altman plot demonstrating good agreement with optimal analysis for these data. (F) Scatter plot demonstrating relationship between R-R_RMT_ and ML-PEST_RMT_ also confirming similarities between estimation tools. Data from participant 12, shown with a triangle, was not included in any statistical analyses.

All fifteen participants successfully completed the study. The difference between the MT estimates was calculated for each individual and assessed for outliers. Following these steps, the difference between MT estimates (R-R and ML-PEST) from one individual was deemed an outlier (>3SD) for both RMT and AMT (participant 12) and was not included in any statistical analyses. The group-averaged number of TMS pulses used to reach AMT with the R-R (R-R_AMT_) and ML-PEST (ML-PEST_AMT_) methods was calculated. As expected, the number of stimuli required to reach AMT was greater with R-R (37.1 ± 4.43 pulses) versus ML-PEST (20 pulses). The group-averaged MSO for AMT obtained by R-R (30.4 ± 3.97% MSO) and ML-PEST (29.9 ± 3.96% MSO) are shown in Fig 1A and were not statistically different (p = 0.26). Fig 1B displays a Bland-Altman plot of individual participant data to provide a visualization of the similarities between the R-R_AMT_ and ML-PEST_AMT_. This plot demonstrates limits of agreement ranging from -3.08% to 4.22% MSO between R-R AMT and ML-PEST AMT. This represents good agreement since the mean difference between the two methods is 0.57 ± 1.82% MSO. This amount of variation in AMT is less than that observed across days for estimates of MT [16]. Additionally, strong positive Pearson correlation was observed between R-R and ML-PEST estimates of AMT (r = 0.89, p < 0.001), and intraclass correlations revealed strong agreement between R-R and ML-PEST estimates (ICC = 0.944, p < 0.001) confirming their similarity in AMT estimation, as shown in Fig 1C.

The number of TMS pulses required to reach RMT with the R-R (R-R_RMT_) and ML-PEST (ML-PEST_RMT_) methods was calculated. As expected, the number of stimuli required to reach RMT was greater with R-R (35.1 ± 8.21 pulses) versus ML-PEST (20 pulses). Fig 1D displays the group-averaged MSO for RMT obtained by R-R (40.6 ± 6.78% MSO) and ML-PEST (40.1 ± 7.30% MSO) which were not significantly different (p = 0.51). Fig 1E displays a Bland-Altman plot of participant data with limits of agreement that range from -5.74% to 6.88% MSO between the two MT estimation methods. This represents good agreement since the mean difference between the two methods is 0.57 ± 3.15% MSO. Similar to AMT, this level of agreement is acceptable as it is less variation than that seen within individuals over time [16, 17].

Additionally, strong positive correlation was observed between R-R and ML-PEST estimates of RMT (r = 0.90, p < 0.001) and intraclass correlations revealed strong agreement between R-R and ML-PEST estimates (ICC = 0.947, p < 0.001) confirming their similarity in RMT estimation, as shown in Fig 1F.

## Discussion

In the present study, the R-R and ML-PEST methods were compared for measures of AMT and RMT in the right FDI muscle in healthy humans. Our data indicates that ML-PEST can estimate AMT with fewer pulses compared to the R-R method, while showing no significant difference in % MSO between R-R and ML-PEST. These novel findings demonstrate that the advantages of ML-PEST over the R-R method for RMT [3, 8] now extend to include the estimation of AMT. Further, we confirm previous observations regarding the efficiency of ML-PEST over R-R approaches for estimation of RMT [7].

The greater efficiency of the ML-PEST approach for AMT may also relate to the starting intensity used for the R-R method (i.e. 37% MSO). The R-R method is sensitive to the starting intensity requiring greater or fewer pulses if the initial intensity is further or closer to the actual AMT, respectively. We used 37% MSO as the initial intensity for R-R method to match that embedded in the ML-PEST software, a value that is closer to RMT versus AMT. Therefore, it is possible that other starting intensities, closer to the actual AMT would reduce the differences observed between the two approaches. However, we note that the sensitivity of the R-R method to the initial starting intensity provides further support for the use of ML-PEST to estimate both AMT and RMT.

The ML-PEST approach requires 20 TMS pulses to yield AMT/RMT, achieving a 95% confidence interval within the accuracy limits imposed by safety guidelines [3, 13, 18]. In the present study, MT was never achieved within 20 or fewer pulses in the R-R method. Specifically, the R-R method required an average of 17 and 15 additional pulses to achieve AMT and RMT, respectively. Despite this difference in the required number of pulses, the estimations obtained using the two methods did not differ statistically for AMT and RMT. Thus, we demonstrate the efficiency of the ML-PEST method for AMT and confirm the same result for measures of RMT. Further, methodological comparison of these two MT estimation techniques using Bland-Altman plots demonstrate that the mean difference between the two methods was less than 1% MSO for both AMT (Fig 1B) and RMT (Fig 1E), demonstrating strong agreement between R-R and ML-PEST protocols. Last, we note that the R-R method, modified for incremental change of 4% rather than the originally published 5% appears to improve the efficiency of the R-R approach. Specifically, Silbert et al. (2010) used the original 5% increment and 56.8 ± 4.3 pulses were required to reach RMT. In the present study, R-R method using 4% increments demonstrated that RMT was achievable in 35.3 ± 8.2 pulses, but was still less efficient by 15 pulses compared to ML-PEST.

We noted that one individual (participant 12) had differences between the two approaches that were deemed outliers (> 3SD). This odd outcome could be potentially explained by extreme hysteresis effect in subject 12, whereby previous activation of the neurons upon TMS delivery during R-R method, which was performed first in this subject, increased excitability of the cells and resulted in lower MT during ML-PEST acquisition [19]. Alternatively, we cannot exclude the possibility that the coil or headband equipped with the subject tracker was inadvertently re-positioned during collection. However, we think this scenario is unlikely since the same experimental practices for coil handling and Neuronavigation were used for all participants.

### Limitations

In the present study, we used a 50 mm TMS coil, and such sized coils are becoming increasingly popular. Since this research replicated the RMT data obtained with the 70 mm coil [7], we anticipate that our AMT results apply to larger diameter coils. Further, the present study was conducted in young adults and it remains to be seen whether similar findings are observed in the elderly that demonstrate age-related declines in RMT and corticospinal excitability [8].

### Conclusion

The present results add to the increasing evidence that favor adaptive threshold-hunting methods for determining MT and provides further empirical support for the recent recommendation of IFCN for MT estimation [3, 8]. Further, due to the rapid acquisition of RMT/AMT using adaptive threshold hunting, this technique is ideally suited to monitor state-dependent changes that occur following plasticity-inducing interventions. For example, threshold hunting can detect rapid changes in RMT that occur with changes in visual attention and motor imagery [20]. In conclusion, accumulating evidence suggests that the use of adaptive threshold hunting methods of MT estimation benefit researchers in that fewer stimuli are required while providing similar outcomes obtained by traditional methods. The efficiency of the ML-PEST approach will be most notable when multiple muscles that each require a measure of AMT and/or RMT are tested within a session. Future studies should explore the effectiveness of ML-PEST methods in other experimental conditions such as following the induction of neuroplasticity and in special populations.
